# Re-expansion pulmonary edema post-pneumothorax

**DOI:** 10.1093/burnst/tkaa032

**Published:** 2020-09-01

**Authors:** He Fang, Long Xu, Feng Zhu, Zhaofan Xia

**Affiliations:** Department of Burn Surgery, Changhai Hospital Affiliated to the Naval Military Medical University, 168 Changhai Road, Yangpu District, Shanghai 200433, China; Department of Burn Surgery, Changhai Hospital Affiliated to the Naval Military Medical University, 168 Changhai Road, Yangpu District, Shanghai 200433, China; Department of Burn Surgery, Changhai Hospital Affiliated to the Naval Military Medical University, 168 Changhai Road, Yangpu District, Shanghai 200433, China; Department of Burn Surgery, Changhai Hospital Affiliated to the Naval Military Medical University, 168 Changhai Road, Yangpu District, Shanghai 200433, China

To the Editor

Re-expansion pulmonary edema (REPE) is an uncommon complication that occurs in patients suffering from lung collapse, especially in cases of extensive and long-term pneumothorax or pleural effusion. After thoracentesis or thoracic tube drainage is carried out, the collapsed lungs can be re-expanded and acute lung edema occurs in unilateral or even bilateral lungs within a short time. Although the reported incidence of REPE is <1% [1], it can have life-threatening consequences, as reported in some studies [2]. In this article, we present the case of a young male patient who developed REPE after suffering traumatic pneumothorax.

A 37-year-old man was transferred to the emergency department with severe destructive injuries, namely a comminuted fracture of the lower right femoral shaft, ruptures of the lower right femoral artery and vein and avulsion of skin and soft tissue in the right lower limb. Eight hours earlier, the patient was hit by a twisted cable rope. The patient was admitted to the burn and trauma intensive care unit. On admission, his vital signs were as follows: heart rate, 88/min; blood pressure, 85/52 mmHg; respiratory rate, 18/min; pulse oximetric saturation (SpO_2_), 99%; and arterial blood partial pressure of oxygen (PaO_2_), 176 mmHg. The chest X-ray was normal (Figure 1a). The laboratory tests showed a hemoglobin level of 72 g/L (normal range: 120–160 g/L) and a hematocrit of 19.5% (normal range: 42–49%). Resuscitative measures were carried out immediately. Despite emergency femoral artery and vein repair surgery being carried out immediately, the patient’s distal lower extremity showed ischemic manifestations 1 day post-surgery. After comprehensive evaluation, including vascular ultrasound and computed tomography angiography, amputation of the right leg above the knee was performed under general anesthesia with endotracheal intubation. On the second day after surgery, the SpO_2_ of the patient was decreased gradually from 100% to 88%. The situation did not improve with increasing oxygen concentration. Blood gas analysis indicated that the PaO_2_ had dropped to 81 mmHg. Physical examination showed right thorax fullness and silent right breath sounds. Rapid chest X-ray showed severe right pneumothorax (Figure 1b). A chest drain was inserted into the right pleural cavity under ultrasound guidance at the intersection of the fourth intercostal line and the right anterior axillary line for continuous closed thoracic drainage (−0.3 kPa), although the cause of pneumothorax was not very clear.

**Figure 1.**
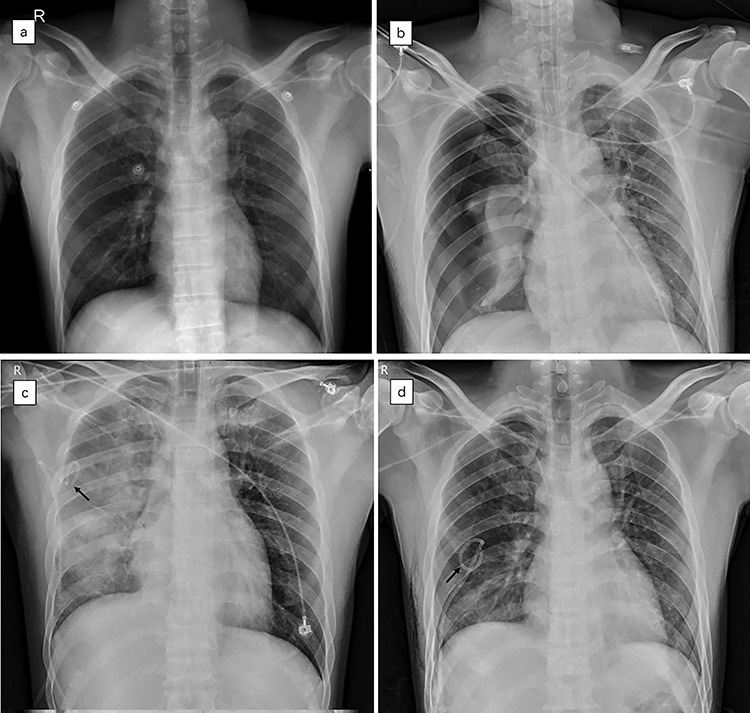
Temporal sequence of chest X-ray imaging of the patient suffering from re-expansion pulmonary edema (REPE). **(a)** Chest X-ray showing normal pulmonary field of both hemithorax after injury. **(b)** Chest X-ray showing large right pneumothorax (estimated 85%) with collapsed right lung and contralateral mediastinal deviation. **(c)** Chest X-ray showing most of the right lung has been re-expanded, accompanied by massive exudative change. **(d)** Chest X-ray showing bilateral clear lungs with free pleural space after 3 days of treatment. The black arrows indicate the position of the chest tube

On the following day, the patient suffered worsening respiratory distress and his SpO_2_ declined. Chest X-ray examination showed successful pulmonary re-expansion and right lung edema (Figure 1c). Based on the clinical and radiological findings, a diagnosis of REPE was made. The patient subsequently received comprehensive treatment measures, including limited fluid input, diuretics, sedation, analgesia and mechanical ventilation with high positive end expiratory pressure (PEEP). His pulmonary function completely recovered 3 days later, and a further chest X-ray showed clear bilateral lung fields (Figure 1d).

REPE is considered an iatrogenic complication occurring in patients who undergo rapid re-expansion of collapsed lungs following pleural effusion and pneumothorax. REPE was first reported after thoracentesis in 1853, and after pneumothorax treatment in 1958 [3]. Since then, there have been several reports of REPE following pleural fluid aspiration and pneumothorax. Although the pathophysiological mechanism of REPE has not yet been clarified, it is generally believed that increased vascular endothelial permeability and destruction of microvessels are the most important potential mechanisms [4]. Lung collapse induced by pleural effusion and pneumothorax can lead to histological abnormalities, such as thickening of the pulmonary microvasculature. When the lung is re-expanded, especially when undergoing a large amount of drainage or rapid drainage, the mechanical stretching of the collapsed lung will injure the pulmonary microvasculature and increase its permeability [5]. This is why it is recommended that the drainage volume of pleural effusion does not exceed 1–2 L every 2 h [6]. Some inflammatory factors, such as interleukin-8 and reactive oxygen species, have been reported to play a role in the pathophysiology of REPE [7]. The role of pulmonary surfactant in REPE is still controversial [8]. In the present case, although the duration of the lung collapse caused by pneumothorax was not long, the range of the pneumothorax was large and the drainage volume was not monitored at the time, and so a serious REPE occurred. In addition, the cause of the patient’s pneumothorax might be related to improper use of mechanical ventilation, so the damage to the microvessels in the lungs was also more serious, which might also be another reason for the patient’s REPE.

The diagnosis of REPE is made mainly based on radiological findings [9]. Radiological examination shows ground-glass opacity or consolidation in the collapsed lung or in the contralateral lung, and this can be accompanied by respiratory symptoms or not. Common symptoms are coughing, chest pain and dyspnea.

Although the reported incidence of REPE is low, it can result in life-threatening consequences, which indicates that we should pay more attention to the prevention of its occurrence. When performing pneumothorax drainage, the drainage tube should be connected to the underwater sealed drainage device, rather than directly connected to the negative-pressure suction device—this will help monitor the drainage speed. During the drainage process, especially in the early hours, the patient’s signs and symptoms should be closely observed. If the patient has cough, chest pain, dyspnea or the oxygen saturation drops again, the drainage should be stopped. When REPE occurs, the treatment is generally conservative and supportive. Depending on the individual’s condition, different measures can be taken, such as oxygen supplementation, diuretic application, endotracheal intubation and mechanical ventilation. It should be noted that the suction must be reserved in case of pneumothorax under mechanical ventilation and must be progressive.

Clinical reports have shown that REPE caused by closed thoracic drainage after pneumothorax is more rare and has no clear correlation with thoracic air volume, drainage velocity or pleural pressure, but its mortality is high [10]. The recommended rate and volume of removal in pneumothorax are unclear, though pneumothorax is thought to be closely related to REPE. One possible reason is that it is difficult to quantify air volume.

In this paper, we systematically described a case of REPE after pneumothorax. The phenomenon of unexplained pneumothorax after trauma is common, but REPE caused by closed thoracic drainage after pneumothorax is rare. As REPE is extremely dangerous and tricky, we should try our best to prevent it and pay attention on the treatment strategy of pneumothorax. Pneumothorax should be treated more carefully than pleural effusion because air drainage is unquantifiable and easily overlooked.

## Abbreviations

REPE: re-expansion pulmonary edema; SpO_2_: pulse oximetric saturation; PaO_2_: arterial blood partial pressure of oxygen

## Funding

This paper was supported by the National Nature Science Foundation of China (81701899), the Youth Incubation Plan of the Military Medical Science and Technology Project (16QNP091) and the Naval Medical University Youth Start-up Fund (2016QN10).

## Availability of data and materials

The patient and all authors agreed to share the data included in this case report.

## Ethics approval and consent to participate

Written consent to publish case report was obtained from the patient, which complies with the regulations of the Ethics Committee of Changhai Hospital.

## Authors’ contributions

HF and LX were responsible for writing and designing the manuscript and producing the figures. LX was responsible for searching the literature. HF were responsible for data interpretation, data analysis and editing the manuscript. ZFX and FZ were responsible for reviewing and providing quality oversight for the manuscript and the figures. Written consent for publication was obtained from the patient.

## Conflicts of interest

The authors declare that they have no competing interests.
